# Supplementary material for: Behavioural movement strategies in cyclic models
Source: Sci Rep. 2021 Mar 19;11:6413. doi: 10.1038/s41598-021-85590-y (PMC7979998; doi:10.1038/s41598-021-85590-y)
Supplement: Supplementary file 1 — Supplementary Information 1. [file 41598_2021_85590_MOESM1_ESM.docx]

**Supplementary Table S1**: Statistical Results for Species Densities as a function of the Perception Radius.

Mean value (<ρ_i_>), Standard Deviation (σ_i_), and Variation Coefficient (cv_i_), with i=0...5, where i=0 represents the empty spaces, and i=1...5 indicate the species i. The results were obtained of 100 simulations of lattices with 500² grid points, for Perception Radius (R) varying from 1 to 5.

| **Standard Model** | | | |
| --- | --- | --- | --- |
|  | < ρ_i_ > | σ_i_ | cv_i_ (%) |
| i=0 | 0.0297353 | 0.000225777 | 0.759289464 |
| i=1 | 0.194042 | 0.00106466 | 0.5486750291 |
| i=2 | 0.193858 | 0.00104639 | 0.5397713791 |
| i=3 | 0.194058 | 0.000861961 | 0.4441769986 |
| i=4 | 0.194058 | 0.000861961 | 0.4441769986 |
| i=5 | 0.194117 | 0.00104644 | 0.5390769484 |
|  |  |  |  |
| **Attack Tactic** | | | |
|  | <ρ_0_> | σ_0_ | cv_0_(%) |
| R=1 | 0.0303483 | 0.000353039 | 1.16329086 |
| R=2 | 0.0325617 | 0.000270635 | 0.8311451798 |
| R=3 | 0.0327265 | 0.000256941 | 0.7851160375 |
| R=4 | 0.0330276 | 0.000291635 | 0.8830039119 |
| R=5 | 0.0333033 | 0.000339929 | 1.020706657 |
|  |  |  |  |
|  | < ρ_1_ > | σ_1_ | cv_1_(%) |
| R=1 | 0.213919 | 0.00229117 | 1.071045583 |
| R=2 | 0.206566 | 0.00171167 | 0.8286310429 |
| R=3 | 0.196668 | 0.00120333 | 0.6118585637 |
| R=4 | 0.188353 | 0.00134636 | 0.7148067724 |
| R=5 | 0.184988 | 0.00139249 | 0.7527461241 |
|  |  |  |  |
|  | <ρ_2_> | σ_2_ | cv_2_(%) |
| R=1 | 0.147167 | 0.00200539 | 1.362662825 |
| R=2 | 0.12951 | 0.00123013 | 0.9498339897 |
| R=3 | 0.126712 | 0.000920398 | 0.726370036 |
| R=4 | 0.128581 | 0.000904225 | 0.7032337593 |
| R=5 | 0.132982 | 0.00111128 | 0.8356619693 |
|  |  |  |  |
|  | <ρ_3_> | σ_3_ | cv_3_(%) |
| R=1 | 0.235828 | 0.00159982 | 0.6783842461 |
| R=2 | 0.282023 | 0.00162894 | 0.5775911894 |
| R=3 | 0.304423 | 0.00155994 | 0.5124251453 |
| R=4 | 0.312964 | 0.00204832 | 0.6544906123 |
| R=5 | 0.312603 | 0.0020103 | 0.6430840395 |
|  |  |  |  |
|  | <ρ_4_> | σ_4_ | cv_4_(%) |
| R=1 | 0.182268 | 0.00115451 | 0.6334134352 |
| R=2 | 0.149478 | 0.000961065 | 0.6429474572 |
| R=3 | 0.131477 | 0.00105348 | 0.8012656206 |
| R=4 | 0.122444 | 0.00147364 | 1.20352161 |
| R=5 | 0.116953 | 0.00141843 | 1.212820535 |
|  |  |  |  |
|  | <ρ_5_> | σ_5_ | cv_5_(%) |
| R=1 | 0.19047 | 0.00214104 | 1.124082533 |
| R=2 | 0.199861 | 0.00140899 | 0.7049849646 |
| R=3 | 0.207993 | 0.00123478 | 0.5936642099 |
| R=4 | 0.21463 | 0.00129602 | 0.6038391651 |
| R=5 | 0.219169 | 0.0013448 | 0.6135904257 |
|  |  |  |  |
| **Anticipation Tactic** | | | |
|  | < ρ_0_ > | σ_0_ | cv_0_(%) |
| R=1 | 0.0297778 | 0.000248212 | 0.8335471391 |
| R=2 | 0.0288628 | 0.000223064 | 0.7728425517 |
| R=3 | 0.0249515 | 0.000307133 | 1.230919985 |
| R=4 | 0.0212796 | 0.000342809 | 1.61097483 |
| R=5 | 0.0183646 | 0.000354898 | 1.932511462 |
|  |  |  |  |
|  | < ρ_1_ > | σ_1_ | cv_1_(%) |
| R=1 | 0.186123 | 0.00121333 | 0.6518968639 |
| R=2 | 0.161224 | 0.00134913 | 0.8368046941 |
| R=3 | 0.161035 | 0.00201103 | 1.248815475 |
| R=4 | 0.163878 | 0.00288341 | 1.759485715 |
| R=5 | 0.161895 | 0.00340153 | 2.101071682 |
|  |  |  |  |
|  | <ρ_2_> | σ_2_ | cv_2_(%) |
| R=1 | 0.203307 | 0.00107939 | 0.530916299 |
| R=2 | 0.23164 | 0.00121278 | 0.5235624245 |
| R=3 | 0.244665 | 0.0023343 | 0.9540800687 |
| R=4 | 0.254966 | 0.00378108 | 1.4829742 |
| R=5 | 0.278134 | 0.00584534 | 2.101627273 |
|  |  |  |  |
|  | <ρ_3_> | σ_3_ | cv_3_(%) |
| R=1 | 0.206013 | 0.00131792 | 0.6397266192 |
| R=2 | 0.191375 | 0.000938508 | 0.4904026127 |
| R=3 | 0.178262 | 0.00136984 | 0.7684419562 |
| R=4 | 0.172165 | 0.00213199 | 1.238341126 |
| R=5 | 0.162814 | 0.00329355 | 2.022891152 |
|  |  |  |  |
|  | <ρ_4_> | σ_4_ | cv_4_(%) |
| R=1 | 0.178697 | 0.00142018 | 0.7947419375 |
| R=2 | 0.198879 | 0.00109669 | 0.5514357976 |
| R=3 | 0.216282 | 0.0016201 | 0.749068346 |
| R=4 | 0.221553 | 0.00199732 | 0.901508894 |
| R=5 | 0.225026 | 0.00310298 | 1.378942878 |
|  |  |  |  |
|  | <ρ_5_> | σ_5_ | cv_5_(%) |
| R=1 | 0.196082 | 0.000990947 | 0.5053737722 |
| R=2 | 0.18802 | 0.00125877 | 0.6694872886 |
| R=3 | 0.174804 | 0.00141063 | 0.8069781012 |
| R=4 | 0.166159 | 0.00159817 | 0.9618317395 |
| R=5 | 0.153767 | 0.0029202 | 1.899107091 |
|  |  |  |  |
| **Safeguard Tactic** | | | |
|  | < ρ_0_ > | σ_0_ | cv_0_(%) |
| R=1 | 0.0290613 | 0.000262351 | 0.9027503931 |
| R=2 | 0.0297897 | 0.000250958 | 0.8424321158 |
| R=3 | 0.0280671 | 0.000394561 | 1.405777583 |
| R=4 | 0.0278767 | 0.000404443 | 1.450828111 |
| R=5 | 0.0294006 | 0.000387046 | 1.316456127 |
|  |  |  |  |
|  | < ρ_1_ > | σ_1_ | cv_1_(%) |
| R=1 | 0.220489 | 0.00193888 | 0.8793545256 |
| R=2 | 0.257771 | 0.00119686 | 0.4643113461 |
| R=3 | 0.285419 | 0.00141178 | 0.4946342045 |
| R=4 | 0.29368 | 0.00136142 | 0.463572596 |
| R=5 | 0.293667 | 0.00131533 | 0.44789847 |
|  |  |  |  |
|  | <ρ_2_> | σ_2_ | cv_2_(%) |
| R=1 | 0.206461 | 0.00272013 | 1.317503064 |
| R=2 | 0.178502 | 0.00134238 | 0.7520251874 |
| R=3 | 0.150154 | 0.0012908 | 0.8596507586 |
| R=4 | 0.143095 | 0.00120163 | 0.8397428282 |
| R=5 | 0.144504 | 0.0010361 | 0.7170043736 |
|  |  |  |  |
|  | <ρ_3_> | σ_3_ | cv_3_(%) |
| R=1 | 0.168103 | 0.00139862 | 0.8320018084 |
| R=2 | 0.150585 | 0.000987572 | 0.6558236212 |
| R=3 | 0.14644 | 0.00121779 | 0.8315965583 |
| R=4 | 0.145218 | 0.00136975 | 0.9432370643 |
| R=5 | 0.142969 | 0.000969594 | 0.6781847813 |
|  |  |  |  |
|  | <ρ_4_> | σ_4_ | cv_4_(%) |
| R=1 | 0.190879 | 0.00137055 | 0.7180203165 |
| R=2 | 0.218286 | 0.00120895 | 0.5538376259 |
| R=3 | 0.233028 | 0.00129564 | 0.5560018539 |
| R=4 | 0.232481 | 0.00130736 | 0.5623513319 |
| R=5 | 0.228067 | 0.00127124 | 0.5573976068 |
|  |  |  |  |
|  | <ρ_5_> | σ_5_ | cv_5_(%) |
| R=1 | 0.185006 | 0.00115102 | 0.622152795 |
| R=2 | 0.165067 | 0.000994461 | 0.6024590015 |
| R=3 | 0.156892 | 0.00102674 | 0.6544246998 |
| R=4 | 0.157649 | 0.00100654 | 0.6384690039 |
| R=5 | 0.161393 | 0.0010829 | 0.67097086 |
