# Supplementary material for: Behavioural movement strategies in cyclic models
Source: Sci Rep. 2021 Mar 19;11:6413. doi: 10.1038/s41598-021-85590-y (PMC7979998; doi:10.1038/s41598-021-85590-y)
Supplement: Supplementary file 2 — Supplementary Information [file 41598_2021_85590_MOESM2_ESM.docx]

**Supplementary Table S2**: Statistical Results for Selection Risks in terms of the Perception Radius.

Mean value (<ζ_i_>), Standard Deviation (σ_i_), and Variation Coefficient (cv_i_), with i=1...5, with i representing the species. The results were obtained of 100 simulations of lattices with 500² grid points, for Perception Radius (R) varying from 1 to 5.

| **Standard Model** | | | |
| --- | --- | --- | --- |
|  | <ζ_i_> | σ_i_ | cv_i_(%) |
| i=1 | 0.0285073 | 0.0002457 | 0.8618844998 |
| i=2 | 0.0284949 | 0.00025342 | 0.8893521297 |
| i=3 | 0.0285238 | 0.000246388 | 0.8637979512 |
| i=4 | 0.0285024 | 0.000224866 | 0.788937072 |
| i=5 | 0.0284946 | 0.000253418 | 0.8893544742 |
|  |  |  |  |
| **Attack Tactic** | | | |
|  | <ζ_1_> | σ_1_ | cv_1_(%) |
| R=1 | 0.0258958 | 0.000409812 | 1.582542343 |
| R=2 | 0.0269324 | 0.000335809 | 1.246858802 |
| R=3 | 0.0282685 | 0.00029127 | 1.030369493 |
| R=4 | 0.0305677 | 0.000371445 | 1.215155213 |
| R=5 | 0.0327698 | 0.000440878 | 1.345378977 |
|  |  |  |  |
|  | <ζ_2_> | σ_2_ | cv_2_(%) |
| R=1 | 0.0358549 | 0.000447116 | 1.247015052 |
| R=2 | 0.0418224 | 0.000364245 | 0.8709328016 |
| R=3 | 0.0419549 | 0.000366098 | 0.8725989098 |
| R=4 | 0.040981 | 0.000445105 | 1.086125278 |
| R=5 | 0.0398575 | 0.000467685 | 1.173392712 |
|  |  |  |  |
|  | <ζ_3_> | σ_3_ | cv_3_(%) |
| R=1 | 0.024914 | 0.000338243 | 1.357642289 |
| R=2 | 0.023314 | 0.000229901 | 0.9861070601 |
| R=3 | 0.0219062 | 0.000169712 | 0.7747213118 |
| R=4 | 0.0213325 | 0.000182435 | 0.8551974687 |
| R=5 | 0.0212114 | 0.000235374 | 1.109658014 |
|  |  |  |  |
|  | <ζ_4_> | σ_4_ | cv_4_(%) |
| R=1 | 0.0323698 | 0.000382461 | 1.181536494 |
| R=2 | 0.0421068 | 0.000419099 | 0.9953237957 |
| R=3 | 0.0465607 | 0.000596887 | 1.281954524 |
| R=4 | 0.0487024 | 0.00078125 | 1.604130392 |
| R=5 | 0.0491709 | 0.000855712 | 1.740281345 |
|  |  |  |  |
|  | <ζ_5_> | σ_5_ | cv_5_(%) |
| R=1 | 0.0292494 | 0.000382016 | 1.306064398 |
| R=2 | 0.0301792 | 0.000231321 | 0.7664914908 |
| R=3 | 0.0298794 | 0.000228214 | 0.763783744 |
| R=4 | 0.0297843 | 0.00025414 | 0.8532683326 |
| R=5 | 0.0298087 | 0.00027002 | 0.9058429251 |
|  |  |  |  |
| **Anticipation Tactic** | | | |
|  | <ζ_1_> | σ_1_ | cv_1_(%) |
| R=1 | 0.0285582 | 0.000324556 | 1.136472187 |
| R=2 | 0.03092 | 0.000351124 | 1.135588616 |
| R=3 | 0.0276437 | 0.000578183 | 2.091554314 |
| R=4 | 0.0237144 | 0.000647724 | 2.731353102 |
| R=5 | 0.0208712 | 0.000677571 | 3.246440071 |
|  |  |  |  |
|  | <ζ_2_> | σ_2_ | cv_2_(%) |
| R=1 | 0.027687 | 0.000203244 | 0.7340773648 |
| R=2 | 0.0238935 | 0.00018498 | 0.7741854479 |
| R=3 | 0.0195748 | 0.000177254 | 0.9055213846 |
| R=4 | 0.0159982 | 0.000206132 | 1.288469953 |
| R=5 | 0.0126998 | 0.000279671 | 2.202168538 |
|  |  |  |  |
|  | <ζ_3_> | σ_3_ | cv_3_(%) |
| R=1 | 0.0262603 | 0.000269827 | 1.027509206 |
| R=2 | 0.0275794 | 0.000246063 | 0.892198525 |
| R=3 | 0.0249999 | 0.000396082 | 1.584334337 |
| R=4 | 0.021978 | 0.000512027 | 2.32972518 |
| R=5 | 0.0201717 | 0.000607441 | 3.011352538 |
|  |  |  |  |
|  | <ζ_4_> | σ_4_ | cv_4_(%) |
| R=1 | 0.0325411 | 0.000239419 | 0.7357434137 |
| R=2 | 0.0298579 | 0.000212775 | 0.7126254693 |
| R=3 | 0.0244669 | 0.00031079 | 1.270246742 |
| R=4 | 0.0205644 | 0.000289583 | 1.408176266 |
| R=5 | 0.0174689 | 0.000310361 | 1.776648787 |
|  |  |  |  |
|  | <ζ_5_> | σ_5_ | cv_5_(%) |
| R=1 | 0.027943 | 0.000284601 | 1.018505529 |
| R=2 | 0.0268174 | 0.000280628 | 1.046439998 |
| R=3 | 0.0246101 | 0.000340472 | 1.383464513 |
| R=4 | 0.022352 | 0.000381949 | 1.70879116 |
| R=5 | 0.0210846 | 0.000517952 | 2.456541741 |
|  |  |  |  |
| **Safeguard Tactic** | | | |
|  | <ζ_1_> | σ_1_ | cv_1_(%) |
| R=1 | 0.0249009 | 0.00026461 | 1.062652354 |
| R=2 | 0.0221439 | 0.000172255 | 0.7778891704 |
| R=3 | 0.0197193 | 0.000261417 | 1.325691074 |
| R=4 | 0.0195628 | 0.000293355 | 1.499555278 |
| R=5 | 0.0208538 | 0.000281577 | 1.350243121 |
|  |  |  |  |
|  | <ζ_2_> | σ_2_ | cv_2_(%) |
| R=1 | 0.0270154 | 0.000449923 | 1.665431569 |
| R=2 | 0.0307321 | 0.000393268 | 1.279665236 |
| R=3 | 0.0323313 | 0.00054994 | 1.700952328 |
| R=4 | 0.0326306 | 0.000557295 | 1.707890753 |
| R=5 | 0.0333613 | 0.000472044 | 1.414944861 |
|  |  |  |  |
|  | <ζ_3_> | σ_3_ | cv_3_(%) |
| R=1 | 0.0321797 | 0.000285664 | 0.8877149259 |
| R=2 | 0.0359908 | 0.000260671 | 0.7242712026 |
| R=3 | 0.0334169 | 0.000554116 | 1.658190915 |
| R=4 | 0.0318031 | 0.000544064 | 1.710726313 |
| R=5 | 0.0325916 | 0.00049093 | 1.506308374 |
|  |  |  |  |
|  | <ζ_4_> | σ_4_ | cv_4_(%) |
| R=1 | 0.0255561 | 0.000338109 | 1.323007032 |
| R=2 | 0.0230742 | 0.000274478 | 1.189545033 |
| R=3 | 0.0209949 | 0.000311536 | 1.483865129 |
| R=4 | 0.0217215 | 0.000332265 | 1.529659554 |
| R=5 | 0.0239692 | 0.000339683 | 1.417164528 |
|  |  |  |  |
|  | <ζ_5_> | σ_5_ | cv_5_(%) |
| R=1 | 0.0306026 | 0.000225244 | 0.7360289649 |
| R=2 | 0.0354871 | 0.000259101 | 0.7301272857 |
| R=3 | 0.035797 | 0.000470695 | 1.31490069 |
| R=4 | 0.0356942 | 0.000437077 | 1.224504261 |
| R=5 | 0.0369195 | 0.000411622 | 1.114917591 |
